# Comparison of Rx-defined morbidity groups and diagnosis- based risk adjusters for predicting healthcare costs in Taiwan

**DOI:** 10.1186/1472-6963-10-126

**Published:** 2010-05-17

**Authors:** Raymond NC Kuo, Mei-Shu Lai

**Affiliations:** 1 Institute of Health Care Organization Administration, College of Public Health, National Taiwan University, Taipei, Taiwan; 2 Institute of Preventive Medicine, College of Public Health, National Taiwan University, Taipei, Taiwan; 3 Center for Health Insurance Research, College of Public Health, National Taiwan University, Taipei, Taiwan

## Abstract

**Background:**

Medication claims are commonly used to calculate the risk adjustment for measuring healthcare cost. The Rx-defined Morbidity Groups (Rx-MG) which combine the use of medication to indicate morbidity have been incorporated into the Adjusted Clinical Groups (ACG) Case Mix System, developed by the Johns Hopkins University. This study aims to verify that the Rx-MG can be used for adjusting risk and for explaining the variations in the healthcare cost in Taiwan.

**Methods:**

The Longitudinal Health Insurance Database 2005 (LHID2005) was used in this study. The year 2006 was chosen as the baseline to predict healthcare cost (medication and total cost) in 2007. The final sample size amounted to 793 239 (81%) enrolees, and excluded any cases with discontinued enrolment. Two different kinds of models were built to predict cost: the concurrent model and the prospective model. The predictors used in the predictive models included age, gender, Aggregated Diagnosis Groups (ADG, diagnosis- defined morbidity groups), and Rx-defined Morbidity Groups. Multivariate OLS regression was used in the cost prediction modelling.

**Results:**

The concurrent model adjusted for Rx-defined Morbidity Groups for total cost, and controlled for age and gender had a better predictive R-square = 0.618, compared to the model adjusted for ADGs (R^2 ^= 0.411). The model combined with Rx-MGs and ADGs performed the best for concurrently predicting total cost (R^2 ^= 0.650). For prospectively predicting total cost, the model combined Rx-MGs and ADGs (R^2 ^= 0.382) performed better than the models adjusted by Rx-MGs (R^2 ^= 0.360) or ADGs (R^2 ^= 0.252) only. Similarly, the concurrent model adjusted for Rx-MGs predicting pharmacy cost had a better performance (R-square = 0.615), than the model adjusted for ADGs (R^2 ^= 0.431). The model combined with Rx-MGs and ADGs performed the best in concurrently as well as prospectively predicting pharmacy cost (R^2 ^= 0.638 and 0.505, respectively). The prospective models showed a remarkable improvement when adjusted by prior cost.

**Conclusions:**

The medication-based Rx-Defined Morbidity Groups was useful in predicting pharmacy cost as well as total cost in Taiwan. Combining the information on medication and diagnosis as adjusters could arguably be the best method for explaining variations in healthcare cost.

## Background

Diagnosis information is commonly used for defining morbidities and for estimating the risk of healthcare utilization. Diagnosis based comorbidity scales and risk adjustment tools, such as the Charlson Comorbidity Index[1], Elixhauser index[2], the Johns Hopkins Adjusted Clinical Group (ACG) case-mix system[3], and the Diagnostic Cost Group Hierarchical Condition Category (DCG/HCC) model[4,5] have been verified for their effective use in adjusting healthcare costs risks [6-11]. Although administrative data seems to be comprehensive, efficient, low cost, and are most likely to prevent several common biases associated with primary data, the accuracy and quality of the diagnosis coding remains suspect [12-15]. Previous studies found that the diagnoses identified by administrative data were highly specific but varied greatly in sensitivity and therefore recommended that all available sources of data (e.g. prescription claims database)should be included in order to overcome the potential limitations that come with a single source of data[15]. Pine et. al. also argued that risk-adjustment based entirely on administrative data is imperfect because these data do not discriminate between comorbidities and complications, and the limited numbers of secondary diagnoses within the data may not properly reflect the sickest patients [16].

Prescription claim data has several additional strengths for capturing morbidity conditions compared to diagnoses data. Healthcare purchasers (insurers) that provide a drug benefit package, claim that prescription data is often more reliable, timely, complete, and less of a gamble than diagnostic data [12,13,17]. In addition, for persons with a stable, well-managed chronic disease, a medication-based risk instrument may capture their health risk even without the diagnosis information reported by the providers [17]. Several medication-based morbidity measures have been developed. The Chronic Disease Score (CDS) developed by a team of physicians, pharmacists, and health services researchers at the Center for Health Studies, Group Health Cooperative of Puget Sound (GHC), is an early model for measuring morbidity conditions based on prescription data [18]. Then Clark et. al. demonstrated an approach to assign empirically derived weights for the CDS [19]. Afterwards, the CDS was revised to incorporate more drugs used for treating diseases and conditions in order to fulfil the needs to measure the health status and the risk of healthcare utilization among different types of populations [12,17,20,21]. Although these medication-based risk adjustment tools have been tested, and were found to be valid in predicting future healthcare utilization, most of these tools incorporate a coding algorithm that is applied in the U.S. (i.e. required medication data contains the U.S. National Drug Codes (NDC) or the American Hospital Formulary Service (AHFS) Drug codes) , which makes studies conducted outside the U.S. operationally cumbersome.

The Johns Hopkins Adjusted Clinical Groups (ACG) system was developed to predict healthcare utilization and costs based on groupings of diagnoses [22-24]. The former version of the ACG system provided the Aggregated Diagnosis Codes (ADGs; 32 diagnosis clusters) and ACGs (mutually exclusive, health status categories defined by morbidity, age, and sex) of a given population based on diagnosis data. Version 7.1 of the ACG system incorporated Rx-defined Morbidity Groups (Rx-MGs) into predictive models. Unlike earlier developed medication-based risk adjustment tools which include medication therapeutic classes to identify any limited chronic diseases or conditions, the Rx-MG algorithm first reduces nearly 90 000 U.S. NDCs to approx. 2700 units, then assigns each medication use into one of the 60 Rx-MGs based on criteria consisting of primary anatomico-physiological system, morbidity differentiation, expected duration, and severity [24,25]. For medication data collected outside the U.S., an international mapping algorithm within the ACG system also performs the Rx-MG assignment based on the WHO Anatomical Therapeutic Chemical (ATC) classification [26]. This feature makes the ACG system stand out from the other medication-based risk adjustment tools in that it can be applied to countries where the medication data contains neither NDC nor AHFS codes.

This study aimed to verify if the Rx-MGs of the Johns Hopkins ACG system could be used for adjusting risk and for explaining the variations in healthcare cost in Taiwan. Previous researches have shown diagnosis-based ADGs to be a valid morbidity measure as well as risk adjust instrument for the NHI claim data in Taiwan [27,28], but the application of Rx-MGs in empirical research remains absent. Although in recent studies the Rx-MGs were tested and found to be valid risk adjusters within predictive models (PMs), nevertheless, those studies are based on the limited ranking of age or populations with selected health conditions [24,29,30]. In the present study we compared the performance of Rx-MGs to ADGs and other diagnosis-based risk adjusters for predicting the (concurrent and prospective) total cost and the medication cost under the NHI. The performance of Rx-MGs models were tested with a sample that can represent the entire population. The fit of these models was also tested by age groups to ensure generalizability.

## Methods

### Risk Adjustment Instruments

Two types of risk adjusters within the Johns Hopkins ACG system were chosen for the present study: the diagnosis-based ADGs and the medication-based Rx-MGs [24]. Studies have found the Elixhauser's comorbidity index to be statistically slightly superior to the Charlson system at adjusting for comorbidity [31,32]. Therefore, the Deyo's Charlson Comorbidity Index (CCI) [33] and the Elixhauser's Index[2] were adopted as competitors to the Rx-MGs. All of the morbidity groups or prescription groups measured by those instruments were treated as dichotomous variables in predictive models. We used the ICD codes cited by Quan et. al. to determine if each of these diagnoses were included in any of the Deyo's CCI or Elixhauser's Index [34]. Instead of using the original coding algorithms, the enhanced ICD-9-CM coding algorithms for Charlson and Elixhauser's index were adopted to solve: (1) discrepancies among coding algorithms for some conditions; (2) inconsistent defining of the 6 shared comorbidities of Deyo's and Elixhauser's original ICD-9-CM coding algorithms.

### Study Populations

Taiwan launched a universal National Health Insurance (NHI) Program on March 1, 1995. As of 2007, 22.60 million of Taiwan's 22.96 million population (98.4%) were enrolled in the NHI program [35]. And, as of December 2008, 18 829 hospitals and healthcare providers (92% of all healthcare facilities in Taiwan) and 4180 pharmacies were contracted by the Bureau of National Health Insurance [36]. The NHI program features universal access to healthcare, healthcare with acceptable quality, comprehensive benefits (inpatient and ambulatory care, dental services, traditional Chinese medicine therapy, surgery, examinations, laboratory tests, prescription medications, nursing care, hospital rooms, preventive services, and certain OTC drugs). These features make the NHI claim data an appropriate source for comparing the performance of diagnosis-based as well as medication-based risk adjustment instruments.

The Longitudinal Health Insurance Database 2005 (LHID2005), which consists of one million out of 25.68 million National Health Insurance enrollees in 2005, was used in this study. The LHID2005 database was derived by the Bureau of National Health Insurance (BNHI), Department of Health and maintained by the National Health Research Institutes (NHRI) so as to make it accessible to scientists in Taiwan for research purposes. The use of the data in this study was reviewed and granted by the NHRI. The data used in this study has no unique patient identifier nor any information that could violate the privacy protection policy. All case IDs required for data linkage were encrypted before being released. There is no significant difference in the gender or age distribution, nor is there an average insured payroll-related amount between the patients in the LHID2005 and the original population [35]. This study chose 2006 as the baseline year to predict healthcare cost (medication and total cost) in 2007. The final sample size was 793 239 (81%) which excludes cases with discontinued enrolment in 2006. Because those cases which were not fully enrolled in the NHI program in 2006 had less opportunity for access to healthcare covered by the NHI, the costs of that group might be under-estimated. To test for model fit, the sample was randomly divided into the estimation (training) sample (476 558; 60%) and the validation (testing) sample (316 681; 40%).

### Data Analysis

The information on the prescriptions in LHID2005 includes outpatients/clinics, inpatients, and contracted pharmacies (community pharmacies). Diagnosis data combined the diagnosis codes derived from inpatient and outpatient/clinic claims. Studies show that the truncation of healthcare expenditures in predictive models provides more stable and more robust estimates than using raw dollars [24,37]. But, the cut-offs used for defining the outliers in those researches ranged in general from 0.5% to 20% [38-43], or were set for a fixed amount by the researchers [17,24]. In the present study, we capped pharmacy cost and the total cost at the top 1% of the cases, which are the maximums of USD 1846 and USD 7538 in 2006 as well as USD 2062 and USD 9446 in 2007, respectively.

The diagnoses derived from the National Health Insurance claim data were entered into the Johns Hopkins ACG system for ADGs assignment. The prescription codes within the claim data were first mapped to the WHO ATC codes, then entered into the Johns Hopkins ACG system for Rx-MGs assignment. For measuring the Charlson Index and the Elixhauser's index, the diagnoses for all cases were first screened by a pre-defined algorithm to improve the specificity of these codes, excluding outpatient diagnoses which were identified as with a same disease/condition but had been reported less than 3 times within the year, or it they all appeared in the same month. The exclusion criteria was not applied for the data which were input in the ACG system because the precise algorithm for assigning each single ICD code to the ADG was not disclosed by the Johns Hopkins ACG team. Another concern was that the ADG categories include acute diseases/conditions that are not included by the Charlson Index and the Elixhauser's index. Therefore, excluding those ICD codes that were reported less than 3 times may underestimate the existing acute diseases/conditions.

Multivariate OLS regression was used in the cost prediction modelling. The risk adjusters used in the predictive models included age, gender, Deyo's CCI, Elixhauser's Index, ADGs, and Rx-MGs. Because previous studies found that prior cost is a comparatively accurate predictor of true costs [44], it was also included for prospective prediction in this study. Because the relationship between prior- and current-year costs may not be strictly linear [45], we also examined a functional form that included a squared term of costs in 2006. There were five alternative models for the concurrent prediction and seven models for the prospective prediction fitted in this study. For concurrent prediction, the first model controlled for age and gender only, and was followed by models including Deyo's CCI, Elixhauser's index, ADGs, and Rx-MGs. The fifth model combined both ADGs and Rx-MGs for comparing models that included only one of these indexes. For prospective prediction, the alternative models included the five for concurrent prediction, as well as added models that were adjusted by prior cost and the square term of prior cost. The coefficients of each morbidity group within the selected indices were estimated from the estimation sample. Then the coefficients, excluding those which were statistically non-significant in each alternative model (see appendix), were applied in the validation sample. The performance of each alternative model was compared by its predictive R-square and mean of absolute prediction error (MAPE) estimated by the validation sample. Another indicator was also provided in which the MAPE is divided by the mean of cost, so that the MAPEs could be compared across the models with different means of cost. The fit of the selected models was also tested by age groups (< 18, 18-64, > = 65) for sensitivity analysis. The pharmacy cost and total cost of each group were capped at the top 1% of the cases.

## Results

### Patient characteristics

As shown in Table 1, the estimation and the validation sample have the same distribution of age, gender, number of Rx-MGs, and healthcare utilizations. There were 11% of cases with zero Rx-MGs in the estimation samples as well as in the validation sample. The average numbers of Rx-MGs for both samples are 7.19 and 7.20. Also, 29% of cases were with more than 10 Rx-MGs. Compared to the year 2006, the mean of the total cost increased by 12% and the mean of the total cost increased by about 10% in 2007.

**Table 1:** Characteristics of estimation and validation samples

	Estimation (n = 476 558) 60%		Validation (n = 316 681) 40%		p-value
	
Characteristics	(n)	(%)	(n)	(%)	
Age					0.641
0-17	105 430	22.1%	70 449	22.2%	
18-44	193 270	40.6%	128 267	40.5%	
45-64	125 831	26.4%	83 385	26.3%	
> = 65	52 027	10.9%	34 580	10.9%	
Mean age (S. D.)	37.3	(21.0)	37.3	(21.0)	
Gender					0.284
Female	241 477	50.7%	160 854	50.8%	
					
Rx_MGs					0.368
0	53 395	11.2%	35 099	11.1%	
< = 3	63 060	13.2%	41 706	13.2%	
< = 6	108 881	22.8%	72 648	22.9%	
< = 9	113 026	23.7%	75 358	23.8%	
> = 10	138 196	29.0%	91 870	29.0%	
Mean number of Rx_MGs (S. D.)	7.19	(4.98)	7.20	(4.97)	0.247
Mean of total cost (USD^a^) (S. D.)					
Y2006	598	(2063.4)	600	(2410.4)	0.626
Y2007	668	(2339.7)	672	(2934.7)	0.575
Mean of pharmacy cost (USD^ a^) (S. D.)					
Y2006	155	(752.2)	158	(1497.9)	0.298
Y2007	169	(808.5)	174	(1916.1)	0.172

a. USD 1. = NTD 32.5

The distribution of each Rx-MG was similar in both samples (see Table 2). A few Rx-MGs had cases less than 1%, and the number of cases for 'Immune disorders' (ALLx040) and 'Cystic fibrosis' (RESx030) were less than 100. Prevalence of several acute diseases/conditions, identified by Rx-MGs, was above 50% among the two samples: 'Allergy/immunology, acute minor', 'Gastrointestinal/hepatic, acute minor', 'Pain and inflammation', 'Infectious, acute minor', and 'Respiratory, acute minor'. The prevalence of all Rx-MGs had no significant differences among the two samples, except for 'Endocrine, Bone disorders'

**Table 2:** Frequency of Rx-MGs in 2006, by study sample

		Estimation (n = 476 558) 60%		Validation (n = 316 681) 40%		p-value
		
Rx-MG label	Description	(n)^a^	(%)	(n)^ a^	(%)	
	**Allergy/immunology**					
ALLx010	Acute minor	252 733	53.00%	168 474	53.20%	0.145
ALLx030	Chronic inflammatory	86 509	18.20%	57 687	18.20%	0.474
ALLx040	Immune disorders	28	< 0.1%	18	< 0.1%	0.913
ALLx050	Transplant	437	0.10%	277	0.10%	0.538
	**Cardiovascular**					
CARx010	Chronic medical	17 517	3.70%	11 817	3.70%	0.197
CARx020	Congestive heart failure	14 754	3.10%	9 956	3.10%	0.229
CARx030	High blood pressure	79 187	16.60%	52 759	16.70%	0.610
CARx040	Hyperlipidemia	21 877	4.60%	14 572	4.60%	0.821
CARx050	Vascular disorders	57 775	12.10%	38 503	12.20%	0.641
	**Ears-nose-throat**					
EARx010	Acute minor	3 653	0.80%	2 424	0.80%	0.956
	**Endocrine**					
ENDx010	Bone disorders	2 088	0.40%	1 492	0.50%	0.032
ENDx020	Chronic medical	17 059	3.60%	11 413	3.60%	0.569
ENDx030	Diabetes with insulin	4 813	1.00%	3 167	1.00%	0.666
ENDx040	Diabetes without insulin	21 991	4.60%	14 634	4.60%	0.892
ENDx050	Thyroid disorders	4 050	0.80%	2 681	0.80%	0.877
	**Eye**					
EYEx010	Acute minor: curative	103 889	21.80%	69 173	21.80%	0.648
EYEx020	Acute minor: palliative	79 750	16.70%	52 753	16.70%	0.371
EYEx030	Glaucoma	31 213	6.50%	20 959	6.60%	0.227
	**Female reproductive**					
FREx010	Hormone regulation	16 101	3.40%	10 643	3.40%	0.667
FREx020	Infertility	2 772	0.60%	1 826	0.60%	0.771
FREx030	Pregnancy and delivery	10 894	2.30%	7 113	2.20%	0.243
	**Gastrointestinal/hepatic**					
GASx010	Acute minor	288 597	60.60%	191 999	60.60%	0.533
GASx020	Chronic liver disease	11 622	2.40%	7 708	2.40%	0.893
GASx030	Chronic stable	140 349	29.50%	93 089	29.40%	0.596
GASx040	Inflammatory bowel disease	918	0.20%	613	0.20%	0.926
GASx050	Pancreatic disorder	33 833	7.10%	22 647	7.20%	0.379
GASx060	Peptic disease	91 510	19.20%	61 344	19.40%	0.062
	**General signs and symptoms**					
GSIx010	Nausea and vomiting	69 209	14.50%	46 281	14.60%	0.257
GSIx020	Pain	108 551	22.80%	72 225	22.80%	0.765
GSIx030	Pain and inflammation	371 310	77.90%	247 044	78.00%	0.316
	**Genitourinary**					
GURx010	Acute minor	27 817	5.80%	18 636	5.90%	0.375
GURx020	Chronic renal failure	1 086	0.20%	666	0.20%	0.102
	**Hematologic**					
HEMx010	Coagulation disorders	35 047	7.40%	23 460	7.40%	0.369
	**Infections**					
INFx010	Acute major	26 329	5.50%	17 191	5.40%	0.065
INFx020	Acute minor	250 114	52.50%	165 757	52.30%	0.217
INFx030	HIV/AIDS	308	0.10%	203	0.10%	0.928
INFx040	Tuberculosis	1 350	0.30%	883	0.30%	0.714
	**Malignancies**					
MALx010	Malignancies	3 176	0.70%	2 074	0.70%	0.535
	**Musculoskeletal**					
MUSx010	Gout	17 046	3.60%	11 181	3.50%	0.277
MUSx020	Inflammatory conditions	515	0.10%	342	0.10%	0.992
	**Neurologic**					
NURx010	Alzheimer's disease	194	< 0.1%	147	< 0.1%	0.230
NURx020	Chronic medical	56 048	11.80%	37 471	11.80%	0.334
NURx030	Migraine headache	5 341	1.10%	3 588	1.10%	0.612
NURx040	Parkinson's disease	11 633	2.40%	7 656	2.40%	0.506
NURx050	Seizure disorder	8 760	1.80%	5 846	1.80%	0.799
	**Psychosocial**					
PSYx010	Attention-deficit disorder	692	0.10%	465	0.10%	0.852
PSYx020	Addiction	2 531	0.50%	1 614	0.50%	0.195
PSYx030	Anxiety	91 748	19.30%	61 452	19.40%	0.091
PSYx040	Depression	19 482	4.10%	12 951	4.10%	0.973
PSYx050	Acute minor	32 618	6.80%	21 680	6.80%	0.979
PSYx060	Chronic unstable	29 899	6.30%	20 190	6.40%	0.069
	**Respiratory**					
RESx010	Acute minor	301 188	63.20%	200 630	63.40%	0.166
RESx020	Chronic medical	29 992	6.30%	19 890	6.30%	0.820
RESx030	Cystic fibrosis	77	< 0.1%	47	< 0.1%	0.646
RESx040	Airway hyper-reactivity	164 532	34.50%	109 696	34.60%	0.295
	**Skin**					
SKNx010	Acne	23 862	5.00%	15 892	5.00%	0.824
SKNx020	Acute and recurrent	179 162	37.60%	119 167	37.60%	0.753
SKNx030	Chronic medical	3 276	0.70%	2 100	0.70%	0.196
	**Toxic effects/adverse effects**					
TOXx010	Acute major	574	0.10%	381	0.10%	0.986
ZZZx000	Other and nonspecific medications	157 388	33.00%	104 784	33.10%	0.564

a Represents the number of cases which has the selected prescriptions contributed to the Rx-MG (multiple counting, since cases are not mutually exclusive in each Rx-MG).

### Performance comparisons among predictive models

The predictive R-squares of five models predicting total cost concurrently ranged from 0.089 to 0.650 (see Table 3). For those models with cost adjusted by diagnosis-based morbidity measures, the ADGs model performed better than others. The Rx-MGs model has a predictive R-square 0.618, which explains the 21% more variance than the ADGs model. The model that combined ADGs and Rx-MGs had the highest predictive R-square (0.650) as well as the lowest MAPE rate (54.6%) among all models. The prospective prediction models had lower predictive R-squares than the concurrent prediction models. All of the seven models explained less than 50% of the variations in the total cost for 2007. Similar to the concurrent prediction models, the prospective prediction model which combined ADGs and Rx-MGs had a predictive R-square (0.382) that was higher than those using either ADGs or Rx-MGs. The MAPE rate was the lowest (75.9%) among all models except for those that included prior cost. The model which included prior cost increased 0.08 in R-square. The model with the square term for prior cost had no considerable improvement in predictive R-square.

**Table 3:** Predictive models for total cost

Morbidity Index	Source of morbidity	Predictors	Model performance - prediction of total cost					
			
			Concurrent (year 2006)			Prospective (year 2007)		
			R^2^	MAPE	MAPE*(%)	R^2^	MAPE	MAPE*(%)
(none)		Age + gender	0.089	505.9	99.5	0.092	582.9	102.9
Deyo's CCI	Diagnosis	Age + gender + CCIs	0.345	409.0	80.5	0.273	496.4	87.6
Elixhauser's Index	Diagnosis	Age + gender + E. Index	0.373	390.8	76.9	0.294	480.3	84.8
ADG	Diagnosis	Age + gender + ADGs	0.411	360.0	70.8	0.252	486.1	85.8
Rx-MG	Medication	Age + gender + Rx-MGs	0.618	297.5	58.5	0.360	448.5	79.1
ADG + Rx-MG	Diagnosis & medication	Age + gender + ADGs + Rx-MGs	0.650	277.4	54.6	0.382	430.4	75.9
ADG + Rx-MG	Diagnosis & medication	Age + gender + ADGs + Rx-MGs + prior total cost				0.465	386.1	68.1
ADG + Rx-MG	Diagnosis & medication	Age + gender + ADGs + Rx-MGs + prior total cost + (prior total cost)^2^				0.465	389.2	68.7

MAPE, mean absolute prediction error; MAPE*, MAPE divided by the mean of cost

As shown in Table 4, the Rx-MGs models also performed better than the diagnosis-based models for predicting medication cost concurrently and prospectively. But, unlike the results of the total cost prediction models, the ADGs models had a lower predictive R-squares and a higher MAPE rate than the model adjusted by Elixhauser's index for predicting medication cost. The models which combined ADGs and Rx-MGs also improved slightly over the model adjusted by Rx-MGs only. The ADGs and Rx-MGs combined model had a remarkable improvement in predictive R-square after adding the predictor of prior medication cost. The predictive R-square seemed to have only a negligible improvement if the square term of prior medication cost was added.

**Table 4:** Predictive models for medication cost

Morbidity Index	Source of morbidity	Predictors	Model performance - prediction of medication cost					
			
			Concurrent (year 2006)			Prospective (year 2007)		
			**R^2^**	**MAPE**	MAPE* (%)	**R^2^**	**MAPE**	MAPE* (%)
(none)		Age + gender	0.151	155.2	118.9	0.153	166.0	119.1
Deyo's CCI	Diagnosis	Age + gender + CCIs	0.426	113.5	87.0	0.366	129.6	93.0
Elixhauser's Index	Diagnosis	Age + gender + E. Index	0.514	97.4	74.7	0.434	115.5	82.9
ADG	Diagnosis	Age + gender + ADGs	0.431	114.4	87.7	0.360	131.2	94.2
Rx-MG	Medication	Age + gender + Rx-MGs	0.615	89.6	68.6	0.485	110.3	79.1
ADG + Rx-MG	Diagnosis & medication	Age + gender + ADGs + Rx-MGs	0.638	85.6	65.6	0.505	106.2	76.2
ADG + Rx-MG	Diagnosis & medication	Age + gender + ADGs + Rx-MGs + prior medication cost				0.684	73.8	53.0
ADG + Rx-MG	Diagnosis & medication	Age + gender + ADGs + Rx-MGs + prior medication cost + (prior medication cost)^2^				0.684	73.7	52.9

MAPE, mean absolute prediction error; MAPE*, MAPE divided by the mean of cost

### Comparing model performance across age groups

The performance of three alternative models was compared across three age groups: < 18, 18-64, > = 65. After being capped at the 99-percentile of costs for all age groups, the result showed that models that applied to all age ranks had the highest predictive R-squares of all other sub-samples (see Table 5). The 18-64 year old age group had the highest predictive R-squares for all alternative models compared to the other two age groups. For all three sub-samples, the performance of the predictive models was similar to the whole sample: the models that were adjusted for prior cost performed the best. The result showed that R-squares for the 'under 18' age group were the lowest among all three sub-samples, implying that the predictive models are not well explained variations of costs within the sample.

**Table 5:** Total cost predictive models for specific age groups

Predictors	Variation explained by model (R^2^)							
	
	Total cost				Medication cost			
	**(all)**	**Age < 18**	**Age 18-64**	**Age > = 65**	**(all)**	**Age < 18**	**Age 18-64**	**Age > = 65**
	
Concurrent prediction (year 2006)								
Age + gender + Rx-MGs	0.618	0.471	0.589	0.552	0.615	0.399	0.571	0.528
Age + gender + ADGs + Rx-MGs	0.650	0.602	0.629	0.570	0.638	0.520	0.600	0.543
								
Prospective prediction (year 2007)								
Age + gender + Rx-MGs	0.360	0.220	0.333	0.268	0.485	0.244	0.446	0.287
Age + gender + ADGs + Rx-MGs	0.382	0.281	0.359	0.278	0.505	0.308	0.472	0.300
Age + gender + ADGs + Rx-MGs + prior cost	0.465	0.359	0.451	0.376	0.684	0.477	0.677	0.442

## Discussion

This study has demonstrated that the Rx-Defined Morbidity Groups are applicable for predicting the total cost and the medication cost in a universal health insurance system. Although a few articles attempted to predict or explain variations of medication use by applying the Johns Hopkins ACG case-mix system, these analytical models are mainly based on diagnosis-based risk adjusters (i.e. the EDCs, ADGs, or ACGs) within the ACG system [10,11,46]. Two recent articles reported studies that had applied the Johns Hopkins ACG system for identifying high-risk patients and predicting healthcare utilization. However, the authors chose predictive models embedded within the ACG system (i.e. the Dx-PM, Rx-PM, and DxRx-PM) instead of adjusting risks by original morbidity groups (i.e. the ADGs or Rx-MGs) [24,47]. Therefore, we believe that the present article is the first one to describe an empirical study using Rx-MGs for healthcare cost prediction as well as comparing the model performance with other diagnosis-based predictive models.

In this study, the model adjusted by Rx-MGs could explain over 60% of the variations for total cost and medication cost in the concurrent year. Clark et. al. used two versions of the Chronic Disease Score to explain variations of total cost, the R-squares for concurrent prediction were 0.09 and 0.19 [19]. Fishman et. al. used the Rx-Risk model to predict healthcare cost, and the validation R-square of that model was 0.0874. They also took sensitivity analyses for cases with patients younger than 18 or older than 18. The R-squares for these two sub-samples were 0.083 and 0.077, respectively [17]. Sales et. al. used Rx-Risk-V, a modification from Rx-Risk for the veteran population, to predict cost. The R-square of the concurrent prediction was 0.202 [48]. Compared to former researches using medication-based morbidity measures to predict cost, the performance of the Rx-MGs model is relatively better than others. This study also found that the Rx-MGs model is applicable to all the different age groups, although the performance varied among these groups. The Rx-MGs model also performed better than other diagnosis-based alternative models in this study. This finding is consistent with other studies which found that prescription data are superior for predicting pharmacy cost [6,24]. However, our study also found that the Rx-MGs model is superior for predicting total cost. One possible explanation for the superior performance of the Rx-MGs model compared to other medication based morbidity measures reported by previous studies is that the NHI pays for almost all prescription drugs, except for those that are very new in the market, expensive, and not yet approved by the Department of Health. Furthermore, this study aggregated prescriptions from outpatients/clinics, inpatients, and community pharmacies. This comprehensive data was intended to help capture all prescription-related morbidities for each case, something that was not done in similar studies. In addition, the Rx-MGs consisted of not only the chronic diseases or conditions, but they also included several acute diseases or syndromes. This feature makes the Rx-MGs stand out from other chronic disease focused instruments (e.g. the Chronic Disease Score) by capturing all possible risks for healthcare utilization. In addition, although the ADGs do capture the diagnoses of acute diseases or syndromes, the number of ADG categories is smaller than that of the Rx-MGs, which might explain why the performance of Rx-MGs models are superior to the ADGs models. Another possible explanation is that the annual medication cost is merely one fourth of the annual total healthcare cost in NHI. Therefore the model that can explain more variations of medication cost is expected to have a better performance for predicting total cost. However, the real cause for the gap in performance between ADGs and Rx-MGs models needs further investigation.

The predictive R-squares of the ADGs models in this study are larger than those reported by two other similar studies which also used Taiwan NHI data [27,38]. These two earlier studies did not enforce the 'full enrolment' criteria as applied in our study. Therefore the disease burden of those cases selected in these two earlier studies may not be equally accessed. Second, we capped the cost at the 99-percentile, which might be the most critical point to explain the improvement in model performance. We conducted another analysis using original cost (without capping the cost) for the prediction models. The result of that analysis showed that age/sex also adjusted for 4% to 5% of the variances, which is quite similar to Lee and Huang's findings[28]. Chang and Weiner also found that after truncating the cost at top 0.5%, the performance of the models improved significantly[38]. After adjusting for prior healthcare utilization, our proposed model combined with Rx-MGs and ADGs out-performed others models for predicting future medication cost, which could explain over 68% of the variations for future medication cost. The findings of this study are similar to the findings of Forrest, et al.'s study which showed that the Combined Diagnostic/Medication Predictive Models (DxRx-PMs) had the highest R-squares for explaining variations of pharmacy charges and total healthcare charges [24]. Other studies have shown that adding diagnosis-based morbidity measures to medication-based models could improve the prediction of total healthcare utilization [6,19,49,50]. However, those findings supported combining those two types of measure to improve cost prediction. On the other hand, Schneeweiss et.al. compared the performance of four diagnosis-based and two medication-based comorbidity scores to predict mortality. They found that while diagnoses-based scores performed better than medication-based scores in predicting future mortality, combining diagnoses and medication-based scores showed an improvement in predicting mortality [49]. The strength of employing all available diagnosis and prescription data is that some potential risk factors may not be captured in a single morbidity measurement, and each morbidity measurement captures different risks. Therefore, combining different morbidity measures in a given predictive model can be more informative than employing just one. Although using more than one morbidity measurement in a single model may raise the concern of multicollinearity, an empirical study showed that there is only a low correlation between different measures [51].

Previous studies have shown that combined prior costs and morbidity measures are important in determining future high cost patients [24,30,41]. Hsu et. al. found that incorporating information of the previous year's drug use or cost into the risk adjustment approach would greatly improve the accuracy of the prediction. They pointed out that drug costs tend to be stable from year to year and are more predictable than other types of medical costs. Therefore, ignoring past costs may result in preventable misallocation of resources and creates a strong incentives for reverse patient selection [45]. The data of our study also support that predictive models combined with Rx-MGs, ADGs, and prior cost performed the best in predicting future cost. However, investigators have argued that this could provide incentives to increase utilization or to favor a specific style of practicing medicine in addition to medical needs. Thus, payment models that include utilization measures among the predictor variables must proceed with caution [41,52].

Compared to other diagnosis-based predictive models, this study has demonstrated that the Rx-MGs model out-performs all other diagnosis-based models in explaining or predicting healthcare utilization. In future applications, the Rx-MGs could be applied for describing and comparing disease patterns among populations. The models which use Rx-MGs alone or combined with ADGs could also be applied for helping local health authorities or case managers to identify high risk populations for disease management programs [24,29,53]. A comprehensive and integrated care delivery system could be provided to those who have a high utilization of healthcare but have a low severity of illness, instead of delivering fragmented acute care to them. The Rx-MGs or other predictive models within the ACG system could also be tested for their efficiency and appropriateness in allocating healthcare resources or setting payment rates by future researchers or policy makers.

There are several limitations to this study. First, we used ADGs and Rx-MGs as risk adjusters for comparing them with two other commonly used morbidity measures. However, the Johns Hopkins ACG system provides prediction models (PMs) which include disease or frailty markers other than ADGs or Rx-MGs, and they have a better performance than the ADGs or other diagnosis-based measures. The PMs were not included as competing models in this study because the 'risk scores' provided by the Dx-PM or Rx-PM as the summary measures of disease burden were provided by the ACG system [24]. Although the more efficient risk adjusters included in the prediction models could be expected to provide the better performance in predicting cost, the performance of those models is somehow hard to compare with other models that are wholly based on morbidity measures (e.g. the Charlson Comorbidity Index). Second, we excluded those cases with discontinued enrolment in 2006 to ensure equality accessibility for healthcare covered by NHI. However, the reasons for the discontinued enrolment in NHI might be very diverse. Thus these cases that were excluded by our study might be high-risk users (e.g. cancer patients at the end-of-life year) or healthy users (e.g. young students studying abroad). Hence the analytical strategy used in this study could limit its generalizability. Another limitation is the approach to treat outliers in this study. Although we capped at the top 1% of costs, those cases with capped costs generally accounted for approximately 25% of the healthcare expenditure. That implies that the predictive models applied to real data cannot perform as well as in this study. Another analysis also found that when applying the predictive models to those high-risk users with actual cost data, the performance of the models declines significantly. This finding seems to suggest that in order to address this issue it might be best to identify and manage those cases by using the risk adjustment instruments, instead of "predicting" their future healthcare utilization [24,29]. The fourth potential limitation in this study is that we failed to incorporate socio-economic status indicators into the predictive models. However, in a recent article the authors argued that adding socioeconomic patient characteristics improves the predictive model only slightly [54]. The information on socio-economic status is quite limited in the NHI database. We carried out another analysis to incorporate household income into the predictive models. The results showed that as a proxy of the socio-economic status it did not have a statistically significant impact on costs.

## Conclusions

This study demonstrated that compared to other diagnosis-based predictive models, the Rx-MGs model out-performs all other models in explaining variations of cost and predicting future healthcare utilization. For countries or regions that routinely collect prescription claim data, the Rx-MGs within the Johns Hopkins ACG case-mix system could be applied to predict future healthcare utilization as well as allocate resources for healthcare.

## Competing interests

The authors declare that they have no competing interests.

## Authors' contributions

KR contributed to the study design, statistical analysis, interpretation, and writing of the manuscript; LM contributed to the directing and coordinating of the study, leading the panel in developing the NHI drug codes to WHO ATC codes for the mapping algorithms, interpretation and the writing of the manuscript. Both authors have read and approved the final manuscript.

## Pre-publication history

The pre-publication history for this paper can be accessed here:

http://www.biomedcentral.com/1472-6963/10/126/prepub
